# Microglial Activation in Traumatic Brain Injury

**DOI:** 10.3389/fnagi.2017.00208

**Published:** 2017-06-28

**Authors:** Cornelius K. Donat, Gregory Scott, Steve M. Gentleman, Magdalena Sastre

**Affiliations:** Division of Brain Sciences, Department of Medicine, Imperial College LondonLondon, United Kingdom

**Keywords:** traumatic brain injury, CCI, microglia, neuroinflammation, TSPO, polarization states

## Abstract

Microglia have a variety of functions in the brain, including synaptic pruning, CNS repair and mediating the immune response against peripheral infection. Microglia rapidly become activated in response to CNS damage. Depending on the nature of the stimulus, microglia can take a number of activation states, which correspond to altered microglia morphology, gene expression and function. It has been reported that early microglia activation following traumatic brain injury (TBI) may contribute to the restoration of homeostasis in the brain. On the other hand, if they remain chronically activated, such cells display a classically activated phenotype, releasing pro-inflammatory molecules, resulting in further tissue damage and contributing potentially to neurodegeneration. However, new evidence suggests that this classification is over-simplistic and the balance of activation states can vary at different points. In this article, we review the role of microglia in TBI, analyzing their distribution, morphology and functional phenotype over time in animal models and in humans. Animal studies have allowed genetic and pharmacological manipulations of microglia activation, in order to define their role. In addition, we describe investigations on the *in vivo* imaging of microglia using translocator protein (TSPO) PET and autoradiography, showing that microglial activation can occur in regions far remote from sites of focal injuries, in humans and animal models of TBI. Finally, we outline some novel potential therapeutic approaches that prime microglia/macrophages toward the beneficial restorative microglial phenotype after TBI.

## Introduction

Traumatic brain injury (TBI) is the biggest cause of death and disability in the under 40s in the developed world (Langlois et al., 2006; Hyder et al., 2007), both in the civilian and military context. The overall majority of TBIs is a single event; however, repeated injuries among soldiers and athletes are associated with the development of Chronic Traumatic Encephalopathy (CTE) although the pathology of CTE can also be seen after single TBI and its causation remains uncertain (Omalu et al., 2011; Goldstein et al., 2012). Survivors often suffer from debilitating cognitive, emotional and physical impairments. This is further aggravated by the constant failure of clinical trials (reviewed in Loane and Faden, 2010; Gruenbaum et al., 2016), leaving patients without any treatment.

Brain injury can trigger neurodegeneration, which is a major determinant of long-term outcome. Head injury is a major risk factor for the development of dementia, suggested by the presence of amyloid-β (Aβ) plaques in around 30% of post-mortem brain tissue from TBI patients (Roberts et al., 1994). Additionally, amyloid plaques have been observed in surgically removed tissue surrounding contusions in brains of survivors of TBI (Ikonomovic et al., 2004; DeKosky et al., 2007). Recently, it has been shown by Positron Emission Tomography (PET) imaging that the distribution of amyloid plaque in TBI survivors overlaps with that in patients with Alzheimer's disease (AD) but also involves the cerebellum, an area of the brain not typically involved in AD (Scott et al., 2016). Other pathological features of AD are also present in TBI, including increased phosphorylated tau and acetylcholine deficiency (Jordan, 2000; Tran et al., 2011; Goldstein et al., 2012; McKee and Daneshvar, 2015; Shin and Dixon, 2015). In particular, repetitive mild TBI leading to CTE is characterized by perivascular tau pathology, which is also irregularly distributed in the depths of cortical sulci. Another AD susceptibility factor is the Apolipoprotein E isoform 4 (ApoE4), which has been linked with exiguous neurological outcome after TBI (Verghese et al., 2011). This has been associated with alterations in the integrity of the blood brain barrier (BBB) in individuals with ApoE4 genotype (Methia et al., 2001; Nishitsuji et al., 2011).

Additionally, TBI has been linked with Parkinson's disease (Jafari et al., 2013), various psychiatric disorders leading to increased risk of suicide, an overall increase in mortality (McMillan et al., 2011) and peripheral immune suppression (Hazeldine et al., 2015). There are currently no neuroprotective drug treatments available and a major challenge is to understand how brain trauma causes neurodegeneration and other long-term impairments.

A common feature of the neurological pathologies developing as a consequence of TBI is that they initiate and are potentiated by an inflammatory response. Microglial activation occurs early after experimental (Chiu et al., 2016) and human TBI (Ramlackhansingh et al., 2011; Johnson et al., 2013) and can persist for years, detectable both *in vivo* and post-mortem. Sites of activation often coincide with neuronal degeneration and axonal abnormality (Maxwell et al., 2010; Giunta et al., 2012). It was hypothesized that the inflammatory response to TBI therefore could be associated with the subsequent development of neurodegenerative disorders. However, new evidence indicates that glial activation may have also reparative/restorative effects. Thus, it is critical to investigate what causes this inflammatory response, what are the consequences for neuronal degeneration and survival and whether this can be modified with anti-inflammatory therapeutic approaches.

In this review, we analyse the role of microglia in TBI patients and animal models, from imaging studies used to visualize changes in microglia activation *in vivo* and *ex vivo* to studies in post-mortem brains of TBI patients and the prospects of therapies targeting microglia activation in TBI.

## Microglia activation states in TBI

TBI occurs when brain structure and physiology are disrupted due to an extrinsic biomechanical insult to the cranium, resulting in neuronal, axonal and vascular damage. In response to TBI, the brain orchestrates a complex immunological tissue reaction similar to ischemic reperfusion injury (Werner and Engelhard, 2007). It was suggested that, as macrophages, microglia can migrate to the site of the injury, in order to establish a protective environment mitigating deleterious consequences of the injury (Faden et al., 2016). The acute function of microglia in response to TBI is to eradicate cellular and molecular debris. Microglial removal of damaged cells is a very important step in the restoration of the normal brain environment. Damaged cells release Danger-associated molecular patterns (DAMPs), which can become potent inflammatory stimuli, resulting in further tissue damage (Solito and Sastre, 2012; Zhang et al., 2012). Moreover, activated microglia are also capable of releasing noxious substances such as pro-inflammatory cytokines, reactive oxygen species (ROS), nitrogen species and excitatory neurotransmitters i.e., glutamate, which exacerbate damage (Kreutzberg, 1996). While pro-inflammatory cytokines are directly deleterious, they also stimulate the release of glutamate from microglia in an autocrine/paracrine fashion. Therefore, depending on the released amount, this can lead to direct neurotoxic effects on neurons, synapses and dendrites, through ionotropic glutamate receptors and interfere with the glutamate buffering ability of astrocytes by inhibiting astrocytic glutamate transporters (Takaki et al., 2012).

It was initially hypothesized that there is a temporary transition in function of the inflammatory milieu, which in the latter phases favors a protracted inflammatory profile associated with chronic microglial activation, precipitating neurological manifestations, although this view has been found to be far too simplistic.

**Figure 1:**
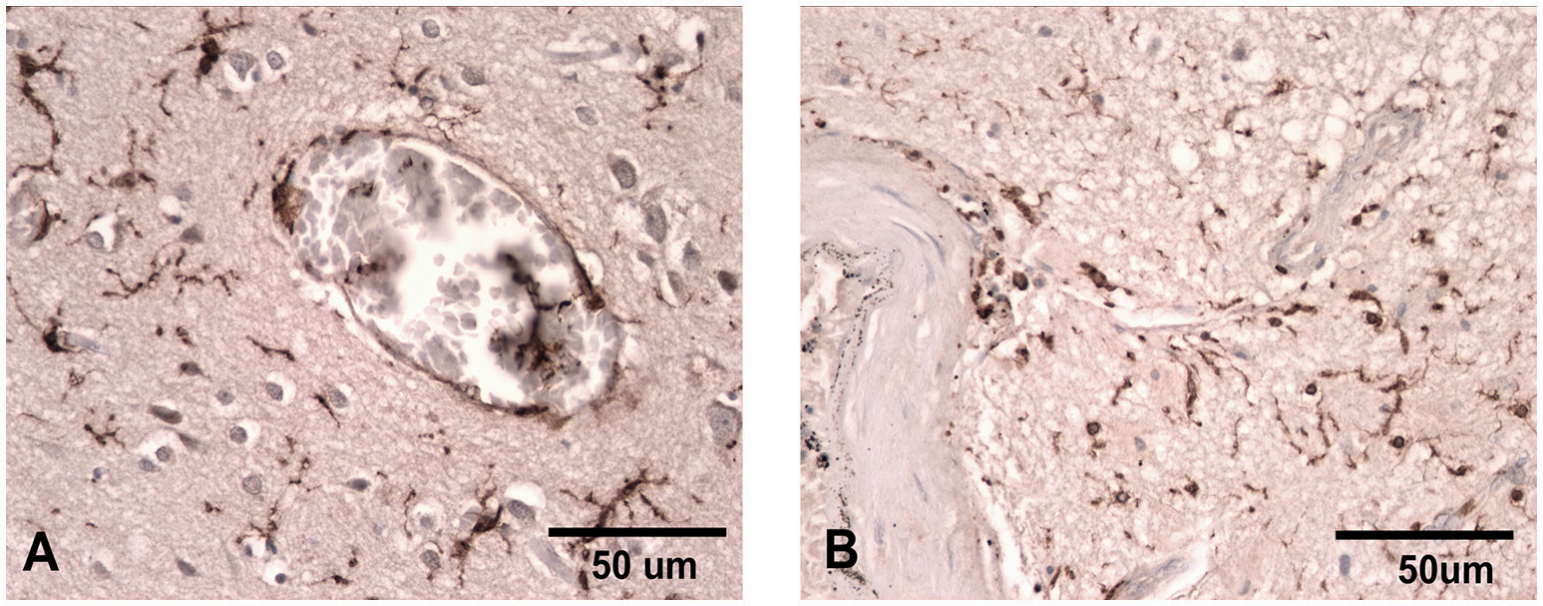
Iba-1 staining illustrating the ramified microglial morphology associated with normal surveillance activity **(A)** compared to that seen in patients where there has been damage to the vasculature and the microglia take on a more rounded phagocytic appearance **(B)**. Scale bar corresponds to 50 μM.

As in other CNS injuries, it seems that microglia activation in TBI results in different phenotypes, corresponding to neurotoxic or neuroprotective priming states. Depending on the stage of the disease and the chronicity, microglia are stimulated differentially and this leads to particular activation states, which correspond to altered microglia morphology, gene expression and function. Microglia are activated in-situ by pro-inflammatory cytokines such as IFN-γ, IL-1α, IL-6, and TNF-α (Ziebell and Morganti-Kossmann, 2010) and become primed. Microglia morphology can switch from a “normal,” ramified shape to a hypertrophic, “bushy” morphology. In response to extensive tissue damage or pathogen invasion, microglia can change into an amoeboid morphology, primarily acting in a phagocytic/macrophage fashion and being difficult to differentiate from infiltrating macrophages (Figure 1). In general, these activation states are classified along a spectrum with M1 or “classically activated” at one end and M2 or “alternatively activated” and the opposite end, similar to macrophages. The proinflammatory M1 phenotype favors the production and release of cytokines that can exacerbate neural injury (Hu et al., 2015). In contrast, the M2 is associated with the release of neurotrophic factors that promote repair and a phagocytic role. However, in recent years, transcriptomic analysis has revealed that microglia and macrophages display a much broader transcriptional repertoire than M1 and M2, depending on the different environmental signals received (Hickman et al., 2013; Xue et al., 2014). Therefore, it is rare to find clear M1 or M2 microglial phenotypes in chronic diseases, since these states are transitory and eventually include mixed activation states. This seems particularly important in TBI as indicated by the many animal studies showing a mixed expression of different markers associated with both M1 and M2 phenotype (Table 1), also described as transitory state (Mtrans) by some authors (Kumar et al., 2016a). Additionally, the inevitable recruitment of bone marrow-derived monocytes into the injured brain parenchyma following experimental TBI and the subsequent differentiation into macrophages complicates the interpretation of the findings, especially with regard to the ability of these cells to change phenotype.

**Table 1:** Microglia/macrophage polarization in experimental models of Traumatic Brain Injury.

**References**	**Species, strain, sex**	**Model, severity and injury details**	**Time post injury**	**Key markers**	**Simplified key results of M1/M2-like marker analysis**
Jin et al., 2012	Mouse C57B6/J ♂	CCI, moderate 3 mm	dpi: 1, 3, 7 14, and 28	**M1**: CD86 **M2**: CD206	**M1**: ▴ 7 + 28 dpi **M2**: ▴ 1 dpi, Sham levels 28 dpi
Bedi et al., 2013	Mouse C57B6 ♂	CCI, moderate ns	dpi: 1	**M1**: FcγRII/III (CD16a/b) **M2**: CD206	**M1/2 ratio**: ▴ at 24 hpi
Walker et al., 2012	Mouse C57B6 ♂	CCI, moderate ns	hpi: 24, 48, 72, 120	**M2**/**M1** ratio of CD206/CD86	**M1**: ▴ 24–72 hpi
Hsieh et al., 2013	Mouse C57BL/6 ♂, YARG/Yet40 knock-in	CCI, moderate 2 mm flat	dpi: 1, 4, 7, 14	**M1**: IL-12p40 **M2**: Arg-1	**M1**: ▴ macrophages 1–14 dpi **M2**: ▴ macrophages 1 dpi Mixed M1/M2 expression
Tchantchou and Zhang, 2013	Mouse C57B6 ♂	CCI, moderate 3 mm flat	dpi: 3,7, 21	**M1**: iNOS **M2**: Arg-1	**M1**: ▴ 3 + 7 dpi, **M2**: no Arg-1
Wang et al., 2013	Mouse C57B6 ♂	CCI, moderate 3 mm flat	dpi: 1, 3, 5, 7, 14	**M1**: CD16/32, iNOS, CD11b, CD86 **M2**: CD206, IL-10, Ym1/2, TGF-β, Arg-1, CCL22	**M1**: ▴ 5–14 dpi **M2**: ▴ 3–5 dpi
Wang et al., 2015	Mouse C57B6 ♂	CCI, moderate 3 mm flat	dpi: 7, 35	**M1**: CD16, iNOS **M2**: CD206, IL-10	**M1**: ▴ 7 dpi **M2**: ▴ 7 dpi
Kumar et al., 2013	Mouse C57B6 ♂,	CCI, severe 3.5 mm flat	dpi: 1, 7	**M1**: IL-1β, TNF-α, CD86, iNOS, CCL2, CCL3 **M2a**: Arg-1, Ym1, CD206/MRC, Fizz-1 **M2c**: IL-4Rα, SOCS3, TGF-β	Mixed M1/M2 expression **M1**: aged > young **M2a**: marker specific ▴▾ **M2c**: young > aged Aged animals ▴ Ym1 on highly activated microglia/macrophages Young animals ▴Ym1 on ramified cells
Loane et al., 2014a	Mouse C57Bl/6 ♂	CCI, severe 3.5 mm flat	wpi: 1, 5, 12, 52	**M1**: MHC II **M2**: Ym-1	M1/MHC II: ▴ 1–52 wpi **M2**: ▴ 1–5 wpi, absent 12–54 wpi
Loane et al., 2014b	Mouse C57Bl/6 ♂	CCI, severe 3.5 mm flat	dpi: 28	**M1**: iNOS **M2**: Arg-1	**M1**: ▴ iNOS/CD11b **M2**: ▾Arg-1/CD11b
Kumar et al., 2016a	Mouse C57Bl/6 ♂	CCI, severe 3.5 mm flat	hpi: 1, 6, 24 dpi: 7	mRNA: **M1**: IL-1β, IL-12, TNF-α, IL-6, iNOS **M2a**: Arg-1, Ym1, CD206, Fizz-1, IL-1rn **M2c**: IL-4Rα, SOCS3, TGF-β Flow cytometry: **M1**: iNOS, IL-12 **M2**: TGF-β, Ym1, CD206, Arg-1 Histology: **M1**: CD16/32, iNOS **Mtrans**: CD16/32+TGF-β, iNOS+, Arg-1 **M2**: TGF-β, Arg-1	Mixed expression of M1/M2 mRNA and protein M1/trans dominate contusion and peri-contusional tissue at 7 dpi
Kumar et al., 2016b	Mouse C57Bl/6 ♂ NOX2 −/−	CCI, severe 3.5 mm flat	dpi: 1, 3, 5, 7, 21, 28	**M1**: IL-1β, NOS2, TNF-α, IL-6, IL-12b **M2**: Arg-1, Ym1, SOCS3, Fizz-1, IL1rn, IL-4Rα **M2c**: IL-4Rα, SOCS3, TGF-β	Mixed expression of M1/2 up to 7 dpi
Morganti et al., 2015	Mouse Dbl-Het C57Bl/6 ♂/♀	CCI, moderate 3 mm convex	hpi: 3, 6, 12 dpi: 1, 2, 7, 14, 28	**M1**: CD68, CD45, MHC II, MARCO, NOS2, TNF-α, CCL2, IL-1β, IL-6, INF-γ **M2a**: Arg-1, Ym1, Fizz1, CD206, IL-4, IL-13 **M2c**: CD36, CD163, TGF-β, IL-10, IL-4Rα, IL-1Ra	Mixed expression of M1 and M2a/c marker mRNA in leucocytes **M1**: ▴ 3 hpi – 14 dpi **M2a**: ▴12 hpi – 7dpi **M2c**: ▴ 6 hpi – 7dpi
Morganti et al., 2016	Mouse C57Bl6/J ♂	CCI, moderate 3 mm convex	dpi: 1, 2, 7	**M1**: CCL2, CCL3, CCL4, CCL6, CCL7, CCL9, CCL12, CCL19, CCL25, CD14, CXCL1, CXCL2, CXCL3, CXCL9, CXCL10, CXCL16, DUSP1, IL-1β, IL-15, IL-16, PTX3, TLR2, TNF-α **M2**: Arg-1, CD36, CX3CL1, IL4R, IL25, TGF-βm TGM2, TLR1, TLR8	Mixed expression of M1 and M2 marker mRNA **M1**: ▴ 3–7 dpi **M2**: ▴ 3–7 dpi Lower fold changes in M2 expression compared to M1
Zanier et al., 2014	Mouse C57Bl/6 ♂	CCI, moderate 3 mm	dpi: 3, 7	**M1**: CD11b, TNF-α, CD68, CD86, IL-1β **M2a/c**: Ym1, Arg-1, CD206, SOCS3, IL-10	Mixed expression of M1 and M2 marker mRNA **M1**: ▴ at 3 + 7 dpi **M2**: ▴ Ym1, Arg-1, SOCS3 at 3 dpi
Febinger et al., 2015	Mouse CX3CR1−/− CX3CR1+/+ ♂	CCI, mild 3 mm	dpi: 7, 15, 30	**M1**: IL-1β, CD86, iNOS, MARCO **M2**: TGF-β, Arg-1, CD206	**M2a**: ▴ Ym1, CD206 in CX3CR1−/− only at 7 dpi ▴ TGF-β in CX3CR1−/− mRNA **M1**: ▾ iNOS, IL-1β 30 dpi: ▴ M1: Marco, CD68
Zanier et al., 2016	Mouse CX3CR1−/− CX3CR1+/+ ♂	CCI, Moderate 3 mm	dpi: 1, 2, 4, 7 wpi: 5	**M1**: CD11b, CD68, iNOS, IL-1β,TNF-α **M2**: Ym1	CX3CR1−/−, 4 dpi: ▴ sensorimotor performance ▾TUNEL positive cells ▾ M1, CD68, iNOS CX3CR1−/− 5 wpi: ▾ sensorimotor performance ▴TUNEL positive cells ▴ M1 iNOS
Desai et al., 2016	Mouse C57BL6/N ♂	CCI, moderate 3 mm flat	hpi: 4 dpi: 1, 4	**M1**: CD16, CD32 **M2**: CD-206, Arg-1, Ym1	**M1**: ▴ CD16, CD32 at 1–4 dpi **M2**: ▴ CD206, Arg-1, Ym1 at 1–4 dpi
Braun et al., 2017	Mouse C57BL/6 CD-1 C3H/OuJ C3H/HeJ CX3CR1 ♂	CCI, severe 3 mm convex	hpi: 24, 72 wpi: 3	**M1**: TNF-α, IL-12 **M2**: TGF-β, IL-10	**M1/M2 ratio**: ▴ 24 + 72 hpi, 3 wpi
Ansari, 2015	Rat Sprague-Dawley ♂	CCI, severe 5 mm flat	hpi: 2, 4, 6, 10, 24	**M1**: TNF-α, IL-1β, IL-6, IFN-γ **M2**: IL-4, IL-10, IL-13, Arg-1, Ym1, Fizz1, MRC-1	Mixed response **M1**: ▴ TNF-α, IL-1β, IL-6 mRNA/protein at 2-10 hpi **M2**: ▴ Arg-1: 6-24 hpi; Ym1: 6 hpi, Fizz1: 4-24 hpi
Turtzo et al., 2014	Rat Wistar ♀	CCI, severe 5 mm flat	dpi: 1, 3, 5, 7, 14, 30 wpi: 8	**M1**: CD40, CD68, TNF-α, CD86, CD80, NOS2 **M2**: CD163, CD206	Mixed expression of M1 and M2 protein expression and mRNA **M1**: ▴CD86 at 5, 7 dpi, TNF-α 1, 3 dpi **M2**: ▴ CD163 at 5, 7 dpi, CD206 at 7 dpi
Barrett et al., 2017	Mouse C57Bl/6 ♂ NOX2 −/−	CCI, severe 3.5 mm flat	dpi: 72	**M2**: IL-4Rα, SOCS3, TGF-β, SHIP1, Arg-1, Ym1, IL-10	NOX2 −/− mice show a robust increase in M2 markers ▴ IL-4Rα, SOCS3, TGF-β, SHIP1, Arg-1, Ym1, IL-10 at 72 hpi vs WT
Cao et al., 2012	Rat Sprague-Dawley ♂	mFPI, moderate 2 atm 4.8 mm craniotomy	dpi: 7, 28	**M1**: TNF-α, CD45 **M2a**: Arg-1 **M2c**: TGF-βI, TGF-βRII	M1 and M2c marker expression Cortex: M1 and M2c: ▴ nonsignificant CD45, TGF-βI, TGF-βRII at 7 dpi, no change in M2a (Arg-1) Thalamus: M1 and M2c: π CD45, TGF-βI, TGF-βRII at 7 and 28 dpi, no change in M2a (Arg-1)
Bachstetter et al., 2013	Mouse p38α MAPK −/− C57BL/6J ♂/♀	mFPI, moderate 1.2 atm 3 mm craniotomy	hpi: 3, 9, 24 dpi 7	**M1**: IL-1β, IL-6, TNF-α, CCL2, CCL5, CXCL1, CD45, CD68, MHC **M2**: Arg-1, Ym1	Mixed expression of M1 and M2 markers mRNA and cytokines/chemokines M1: ▴ IL-6, TNF-α, CCL3 3, 9 hpi, elevated at 7 dpi M2: ▴ Arg-1, Ym1 9 hpi, ▾ below sham levels at 7 dpi, Rod-like microglia p38α MAPK −/−: ▴ M1 and M2 response (mRNA/protein) at 6 hpi ▴ motor performance ▾ Synaptic loss, microglia activation
Fenn et al., 2014	Mouse BALB/c ♂	mFPI, moderate 1.2 -1.5 atm 3 mm craniotomy	hpi: 4, 72	**M1**: IL-1β, CD14, TNF-α, iNOS, IFN-γ, CCL2 **M2**: Arg-1, IL-4Rα, IGF-1, IL-10, IL4	Mixed mRNA expression in ipsilateral cortex and hippocampus, M2 markers less expressed at 72 hpi Cortex, 4 hpi: M1: ▴ IL-1β, CD14, TNF-α, CCL2; M2: ▴ Arg-1, IGF-1 72 hpi: M1: ▴ IFN-γ, CCL2, iNOS; M2: ▴ IL4 Hippocampus, 4 hpi: M1: ▴ IL-1β, CD14, TNF-α, CCL2; M2: ▴ IL-4, IL-10 72 hpi: M1: ▴ IL-1β, CD14, TNF-α, iNOS; M2 ▴ IL-10 ▴ Primed microglia
Fenn et al., 2015	Mouse BALB/c ♂	mFPI, moderate 1.2 -1.5 atm 3 mm craniotomy	dpi: 1	**M1**: IL-1β, CD14, TNF-α, iNOS **M2**: Arg-1, IL-10	Mixed expression at 1 dpi Hippocampus: **M1**: ▴ IL-1β, CD14, TNF-α, CCL2; **M2**: ▴ Arg-1
					Whole brain CD11b positive cells: **M1**: ▴ IL-1β, CD14, TNF-α, CCL2, iNOS; **M2**: ▴ Arg-1
Truettner et al., 2016	Rat Sprague-Dawley ♂	mFPI, moderate 1.8–2.2 atm 4.8 mm craniotomy	hpi: 4, 24	**M1**: iNOS, IL-1β, IL-1α, TNF-α, IL-6. IL-12, CCL2 **M2**: Arg-1, CD163, CD206, IL-10, Ym1, TGF- β **M1/M2**: iNOS/Arg-1 ratio	Mixed expression of M1 and M2 mRNA at 4 and 24 hpi, M1 more pronounced ▴ M1 ratio at 4 h, similar M1/M2 ratio at 24 h in microglia mRNA 4 h: **M1**: ▴ all; **M2**: ▴ (lower relative expression) mRNA 24 h: **M1**: ▴ TNF-α ▴ CD206, TGF-β
Chhor et al., 2016	Mouse OF-1 Postnatal day 7 ♂/♀	WD, closed 2 mm footplate 10 g weight dropped 10 cm	hpi: 2, 6, 14, 24 dpi: 5	**M1** (Cytotoxic): IL-1 α, IL-1β, IL-3, IL-6, IL-9, IL-12p40, IL-12p70, IL-17, INF-γ, TNF-α, CXCL1, CCL3, CCL4, CCL5, Cox-2, iNOS, CD32, CD86 **M2a** (Reparative/regenerative): IL4, IL-13, G-CSF, CCL2, Arg-1, CD206, IGF-1, Gal-3 **M2c** (Immunomodulatory): IL-5, IL-10, SphK1, IL-1rn, SOCS3, IL-4ra	Moderate mixed increase in M1 and M2a/c mRNA Ipsilateral hemisphere: **M1**: ▴ all markers except TNF-α and IL-12(p70) between 6, 14 and 24 hpi **M2a/c**: ▴ all markers except TNF-α and IL-12(p70) between 6, 14 and 24 hpi CD11b+ isolated microglia/macrophages: **M1**: ▴CD32/CD86 up to 1 dpi **M2a/c**: ▴Arg-1, CD206, SOCS3, IL-1rn, Gal3 up to 1 dpi, IGF-1 at 5 dpi Pronounced contralateral effects
Semple et al., 2010	Mouse CCL2 −/− C57Bl/6 ♂	WD, closed 333 g weight dropped 2 cm, silicone tip	hpi: 2, 4, 12, 24 dpi: 4, 7, 14, 28	**M1**: IL-1α, IL-1β, IL-6, IL-12p40, IL-12p70, CCL3, CXCL1, CXCL2, CCL5, TNF-α, INF-γ, **M2a**: G-CSF, IL-10	Primarily M1 driven response **M1**: ▴ IL-1α, IL-6, IL-12p40, CCL5 at 12 hpi **M2**: ▴ G-CSF1, IL-10 only at 12 hpi in CCL2 −/− CCL2 −/−: ▾lesion volume, macrophage accumulation and astrocyte activation at 14, 28 dpi

*Levels of severity for CCI: Mild: 0.5 mm, Moderate: 0.95–1.5 mm; Severe: >2.0 mm*.

*CCI, Controlled Cortical Impact; mFPI, midline Fluid Percussion Injury, WD, Weight drop*.

*Dpi, Days post injury; hpi, hours post injury; wpi, weeks post injury*.

Additional complications arise from the clinical observation that outcome after TBI can be different between male and female adults and post-pubescent adolescents in some but not all studies (Coimbra et al., 2003), with females having lower mortality and less complications (Phelan et al., 2007; Berry et al., 2009; Ley et al., 2013). This can be attributed, at least partially, to astrocytes and microglia, as recently reviewed (Caplan et al., 2017). Gonadal steroid hormones such as progesterone and estradiol are directly involved in the glial response under injury conditions in and during development (Lenz and McCarthy, 2015). Moreover, as progesterone can shift macrophage polarization from M1 to M2 phenotype (Menzies et al., 2011), is seems possible that similar effects could occur in microglia as well, whether the concept of microglia polarization is accepted or not.

The polarization states of microglia could therefore be relevant in the progression of TBI into other neurological disorders such as AD and could interfere with the recovery of the patients and the effectiveness of particular anti-inflammatory treatment.

In addition, the role of infiltrating macrophages and other immune cells are also relevant in the context of microglia dynamics; injury type and parameters may play an important role, with focal models of TBI generally eliciting a more pronounced and localized inflammatory response due to significant tissue damage and pronounced infiltration of peripheral immune cells through the compromised BBB. In line with this, factors that have been implicated in BBB breakdown after injury, such as ApoE (mentioned above) and the extracellular enzymes matrix metalloproteinases (MMPs) have been found to contribute to contusion expansion and vasogenic edema after TBI (Guilfoyle et al., 2015).

## Microglial activation in animal models of TBI

In the past five decades, several animal models have been developed to characterize the biomechanical and pathophysiological patterns of TBI and to provide reliably ways to test treatment strategies. These models almost exclusively employ a single-hit injury and only in the past decade repeated injury models emerged that try to mimic the effect of repetitive injuries. The different animal models address distinct pathophysiological aspects of TBI and have different etiological and construct validity (O'Connor et al., 2011). The most widely applied models of TBI are open-head models, that induce an injury via the intact dura mater upon the underlying cortical tissue (reviewed in Xiong et al., 2013), with focal, diffuse and/or mixed injury patterns, depending on the type of injury. In order to do so, rigid impactors (Controlled Cortical Impact, CCI; weight drop, WD), fluidic pressure (Fluid Percussion Injury, FPI) and blast waves are employed. Additionally, high-velocity probes or projectiles are used to model penetrating brain injury. The CCI, FPI and the various WD models have a long history and were extensively characterized in the past, including assessment of long-term outcome (Kochanek et al., 2002; Immonen et al., 2009).

With the recognition that multiple head injuries, even of sub-threshold severity, are connected to chronic sequel, such as CTE (McKee et al., 2016; Vile and Atkinson, 2017), new models were developed to account for those findings. A growing number of studies in repeated injury rodent models (Shitaka et al., 2011; Klemenhagen et al., 2013; Mannix et al., 2014; Semple et al., 2016; Robinson et al., 2017) indicate that the glial response, in particular increases in microglia numbers and changes in morphology, shares an overall similar pattern with the traditional single-injury models. Interestingly, a few studies showed that repeated injuries can exacerbate the glial response, e.g., microglia or astrocyte cell density, as compared to a single injury (Ojo et al., 2013; Petraglia et al., 2014; Xu et al., 2016; Gao et al., 2017; Tyburski et al., 2017). Recent findings in human post-mortem tissue support the overall notion that repeated injuries result in a stronger glial response by showing that repeated head injury was a predictor of CD68 cell density and phosphorylated tau (Cherry et al., 2016).

The involvement of microglia in the complex pathophysiological cascade following brain injury has long been recognized. Shortly after the description of microglia by Del Rio-Hortega, accumulation of “microglia-like” cells were reported in the tissue around autologous blood injections and a cortical stab wound (Carmichael, 1929; Dunning and Stevenson, 1934) in the rabbit. Similar to reactive astrocytes, microglia are versatile cells, which is exemplified by their heterogeneous morphology (Boche et al., 2013). Perivascular microglia respond to pressure on the thinned mouse skull (Roth et al., 2014) by changing into jellyfish morphology; CCI causes some microglia to align with axon initial segment, the site where action potentials are generated (Baalman et al., 2015) and midline FPI yields several rod-like microglia (Ziebell et al., 2012).

Depending on the employed markers and methods, significantly increasing microglia activation in animal models of TBI is usually apparent from day 1 to 3 post-injury (p.i.) (Bye et al., 2007; Elliott et al., 2011) and can persist chronically beyond 28 days p.i., a usual cut-off time-point for many animal studies. A few studies have elegantly emphasized the long-term nature of microglia activation after experimental TBI, primarily with immunohistochemical analysis showing that major histocompatibility complex (MHC)-II positive cells were still present in the ipsilateral hemisphere at 3 months after WD injury (Holmin and Mathiesen, 1999). Recently, a significant loss of ramified, but increase of hypertrophic microglia in the injured rodent cortex at 1 year after moderate CCI in mice was demonstrated (Loane et al., 2014a). This suggests that, although microglial activation occurs early after TBI, it can change the phenotype and function over time (see Table 1).

### Manipulation of microglial activation in animal models of TBI

Several studies have investigated the effects of microglial elimination in brain injury models, in order to understand their function and the potential as therapeutic approach. The use of the transgenic CD11b-HSVTK mice in response to ganciclovir (GCV) treatment allowed the study of the depletion of proliferating microglia in models of brain or peripheral nerve injury. These transgenic mice express a mutant form of the gene herpes simplex virus 1 thymidine kinase (HSV-1 TKmt-30) driven by the myeloid specific promoter CD11b. HSV-1 TK is capable of phosphorylating specific nucleoside analogs such as GCV, which lead to inhibition of DNA synthesis and cell death during cell proliferation (Cheng et al., 1983; Faulds and Heel, 1990; Black et al., 1996).

Unexpectedly, eliminating proliferative CD11b positive cells and therefore microglia and potentially macrophages in a model of hypoglossal nerve axotomy did not result in pronounced changes in motor neuron loss (Gowing et al., 2006). In another study, employing a repeated closed-head injury in the CD11b-TK mice, low dose of GCV reduced the microglial population after TBI but did not alter the extent of axonal injury as visualized by silver staining. Additionally, higher doses were found to be toxic and aggravated the TBI induced damage (Bennett and Brody, 2014).

Rapid elimination of ~95% of all microglia can be achieved by administration of the colony stimulating factor 1 receptor (CSF1R) inhibitor PLX3397. This approach was used in a model of neuronal injury (Rice et al., 2015) consisting of a diphtheria toxin-induced neuronal lesion in transgenic mice carrying a transgene for the diphtheria toxin A-chain. The diphtheria toxin was activated in transgenic mice by feeding them food without doxycycline, triggering neurotoxicity. The treatment with PLX3397 had different outcomes depending on whether microglia elimination took place in the recovery period only or was administered during the lesion as well as the recovery period. Elimination of microglia after the lesion resulted in improved functional recovery, while microglia depletion during the insult led to greater neuronal loss (Rice et al., 2015). Therefore, in models of CNS damage, it seems that chronically activated microglia display a pro-inflammatory phenotype that may contribute to further synaptic loss. In addition, using the same approach of treatment with the CSF1R inhibitor, it was recently shown that microglia are involved in changes in neuronal network activity and spreading depolarization after excitotoxic lesions and ischemia. Depletion of microglia led to dysregulated neuronal calcium responses, calcium overload and increased neuronal death and infarct size (Szalay et al., 2016), with similar reports in a mouse model of transient focal cerebral ischemia and reperfusion (Jin et al., 2017).

From these findings, it seems obvious that general ablation of microglia is not a productive strategy and perhaps it would be more effective to suppress a specific phenotype at a particular time-point. One approach would be to characterize the different microglial functions based on morphology and phenotype markers in order to find the right time for a microglia-targeting therapeutic strategy.

### Polarization of microglia in TBI models

The polarization of microglia is a relatively new concept, not seen without controversy (Ransohoff, 2016), which has been already investigated in a number of studies, primarily in the CCI model in mice. Only a few studies employed rats or other established TBI models, such as FPI and WD. Data from clinically relevant repeated and penetrating models is lacking even for rodents. To the knowledge of the authors, no study has investigated microglia polarization in gyrencephalic models such as ferrets, sheep or pigs, even though these species had previously been employed in CCI, FPI, penetrating, and blast models.

In the established rodent models (see Table 1), data indicates that microglia and infiltrating macrophages, responding to injury, do not lean toward an extreme end of the spectrum, as shown in several studies through the expression of both M1 and M2-like markers in the “acute” phase. However, it seems that in the subsequent “subacute” and “chronic” phase, the expression of M2-like markers is reduced, while M1-like markers are still expressed, indicating that the pro-inflammatory action of microglia/macrophages exacerbates the pathology. In contrast, studies in non-human primates suggest a trophic role for chronically activated microglia after TBI, indicating a restorative phenotype in the chronic phase (Nagamoto-Combs et al., 2007, 2010).

Another issue is the apparent differences in the expression of murine and human markers of potential microglia polarization, which have been found for macrophages (reviewed in Murray and Wynn, 2011). However, the employed cellular markers of M1-like and the different M2-like microglia/macrophages are not always clearly indicative of the presumed phenotype, which calls for new and more specific markers in murine models (Jablonski et al., 2015). Additionally, mRNA extraction of brain homogenates does not always yield reliable information on the M1/2-like spectrum. Ideally, microglia and macrophages are to be separated by flow cytometry and then assayed for their activation spectrum using transcriptomic analysis (Hickman et al., 2013; Xue et al., 2014).

The studies that employed different therapeutic strategies show that an increase in M2-like markers is often associated with a better cognitive and histopathological outcome (Table 2). It seems therefore imperative for future studies targeting microglia polarization as therapeutic strategies, to assess several markers in the target cells within a sufficient temporal window in order to show a long-term positive outcome.

**Table 2:** Treatments in experimental models of TBI targeting inflammation and microglia.

**References**	**Species, Strain, sex**	**Treatment**	**Injury**	**Simplified treatment outcomes**
Thal et al., 2011	Mouse C57BL6/CrlN ♂	Pioglitazone, rosiglitazone, PPARγ agonists	CCI	Pioglitazone: 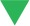 Lesion volume at 24 hpi **M1** 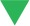 : iNOS, TNF-α, IL-1β mRNA at 24 hpi No beneficial effect of rosiglitazone
Besson et al., 2005	Rat Sprague-Dawley ♂	Fenofibrate, PPARα agonist	FPI	24 hpi: 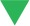 Neurologic deficits 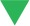 Oedema 7 dpi: 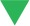 Neurologic deficits
Bye et al., 2007	Mouse C57BL6 ♂	Minocycline	Closed-head WD	4 hpi: 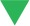 IL-1 β 1 dpi: 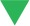 Lesion volume 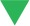 Activated microglia accumulation (F4/80) 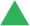 Motor performance 4 dpi: Beneficial effects on lesion size and motor function loss No effect on apoptotic cell death, neutrophil infiltration
Homsi et al., 2010	Mouse Swiss ♂	Minocycline	Closed-head WD	1 dpi: 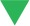 Lesion volume 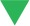 Microglia/macrophages (CD11b) 2 dpi -12 wpi: 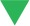 Hyper-locomotor activity 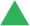 Body weight
Kovesdi et al., 2012	Rat Sprague-Dawley ♂	Minocycline	Blast injury, mild	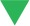 Serum biomarkers of inflammation, vascular and neuronal injury at 51 dpi 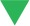 Brain biomarkers in multiple brain regions at 51 dpi 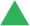 Locomotor activity, spatial memory 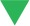 Anxiety
Hanlon et al., 2016	Rat PD 11 Sprague-Dawley ♂/♀	Minocycline	Closed-head CCI, repeated	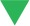 Microglia/macrophages (ED1) accumulation at 3 dpi, Effect lost at 7 dpi, trend toward increase at 21 dpi 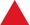 Spatial memory vs vehicle No effect: Iba1 cells, axonal injury, white matter loss and neurodegeneration
Hanlon et al., 2017	Rat PD 11 Sprague-Dawley ♂/♀	Minocycline	Closed-head CCI	Short-term treatment (3 days): 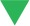 Microglia reactivity in cortex, hippocampus and white matter at 3 but not 7 dpi 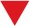 Neurodegeneration at 3 but not 7 dpi Long-term treatment (9 days): 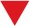 Microglial reactivity and neurodegeneration up to 15 dpi in cortex and hippocampus 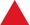 Spatial memory vs vehicle No effects of sex
Chhor et al., 2016	Mouse OF-1 Postnatal day 7 ♂/♀	Minocycline	Closed-head WD	24 hpi: **M1**: 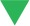 IL-1β, IL-6 **M2a/c**: 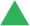 IGF-1, IL-1rn 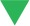 Ventricular volume 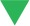 Cleaved caspase 3 positive cells in cortex, hippocampus and striatum 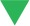 Iba1 positive cells in cortex but not hippocampus/striatum No beneficial effects at 5 dpi
Lloyd et al., 2008	Mouse CD-1 ♂	Minozac	Closed-head CCI	12 hpi: **M1** 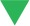 : cytokines levels, IL-1β, IL-6, TNF-α, CCL2 in hippocampus and cortex 28 dpi: 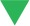 Astrogliosis 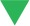 Exploratory deficits
Tchantchou and Zhang, 2013	Mouse C57B6 ♂	WWL70, alpha/beta hydrolase domain 6 inhibitor	CCI	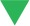 iNOS 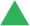 ARG-1 staining 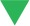 BBB disruption, lesion size, neurodegeneration 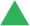 Motor/working memory performance
Loane et al., 2014b	Mouse C57Bl/6 ♂	VU0360172, mGlu5 positive allosteric modulator	CCI	**M1**: 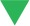 iNOS/CD11b **M2**: 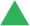 Arg-1/CD11b 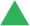 motor performance 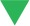 lesion size, neurodegeneration
Wang et al., 2015	Mouse C57B6 ♂	Scriptaid, class I/II histone deacetylase inhibitor	CCI	**M1**: 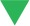 CD16 cells 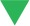 CD16+iNOS mRNA **M2**: 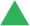 CD206 cells 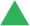 CD206 mRNA 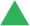 Sensorimotor performance 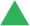 White matter integrity
Morganti et al., 2015	Mouse Dbl-Het C57Bl/6 ♂/♀	CCX872, CCR2 selective antagonist	CCI	Acute phase: **M1** 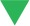 : CD68, CD45, CCL2, IL-1β, IL-6 **M2a** ▾: Arg-1, Fizz1 **M2c** ▾: TGF-β and IL-10 Chronic phase: **M1** 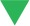 : CD68, CD45, CCL2, IL-1β, TNF-α **M2a** ▾: Fizz1 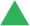 Spatial learning/memory 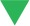 Macrophage invasion
Kumar et al., 2016a,b	Mouse C57Bl/6 NOX −/−♂	gp91ds-tat, selective NOX2 inhibitor	CCI	**M1**: 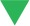 CD16/32 **M2**: 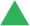 TGF-β **M1**: 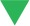 Nox2 **M2**: 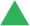 Ym1, Arg-1 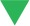 lesion size, neurodegeneration 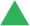 motor/spatial memory performance
Cao et al., 2012	Rat Sprague-Dawley ♂	Ibuprofen	FPI	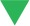 TGF-βI nonsignificant 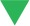 in TSPO, CD45 at 7 dpi in the thalamus
Chio et al., 2013; Cheong et al., 2013	Rat Sprague-Dawley ♂	Etanercept (TNF-α antagonist)	FPI	3/7 dpi: 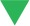 Neurological severity score 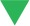 Iba1/TNF-α positive cells in multiple brain regions 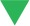 TNF-α protein levels 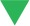 Oedema 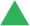 Neurogenesis

*CCI, Controlled Cortical Impact; FPI, Fluid percussion injury, WD, Weight drop*.

Dpi, Days post injury, hpi, hours post injury; wpi, weeks post injury;

*Green color indicates beneficial, while red indicates detrimental actions of the treatment*.

## Imaging of microglia activation in TBI

Measurement of microglial activation *in vivo* has become possible using PET and Single Photon Emission Computed Tomography (SPECT) due to the development of radioligands that bind to the 18-kDa translocator protein (TSPO). TSPO is a five-transmembrane domain protein localized in the outer mitochondrial membrane (Jaremko et al., 2014). The best studied putative function of TSPO relates to its role in transporting cholesterol into the mitochondrial inner membrane space, the rate-limiting step of steroid and neurosteroid biosynthesis (Papadopoulos et al., 2006). However, this function has been lately questioned due to the findings from knockout mice that did not show abnormal steroidogenesis (Morohaku et al., 2014; Tu et al., 2014). Despite almost 40 years of study, the precise functional role of TSPO is far from clear (Selvaraj and Stocco, 2015).

### Imaging of activated microglia after experimental TBI

Autoradiographic studies and PET using TSPO ligands in experimental TBI have informed our understanding of the time-course and spatial distribution of microglial activation following brain trauma. The first TSPO ligand, PK11195, was synthesized in the 1980s. Autoradiographic studies localized binding of radiolabelled PK11195 to activated microglia (Banati et al., 1997) and, as a result, [^11^C]PK11195 was adopted as a PET radioligand to image neuroinflammation *in vivo* (Liu et al., 2014). Increased TSPO-specific radioligand binding was detected as early as 6 h after CCI in rats, with significantly elevated radioligand binding in the ipsilateral and contralateral cortices at 24 h and delayed thalamic upregulation at 28 days p.i. (Donat et al., 2016). Additionally, we previously observed a small increase of [^3^H]PK11195 binding in several brain regions of newborn piglets subjected to FPI at 6 h p.i. (Donat et al., 2014). Similarly, increased TSPO up-regulation indicative of microglia activation was reported in models including FPI (Yu et al., 2010; Cao et al., 2012), CCI (Venneti et al., 2007; Folkersma et al., 2011b; Wang et al., 2014), penetrating brain injury (Miyazawa et al., 1995; Grossman et al., 2012), closed-head injury (CHI) (Grossman et al., 2003) and dynamic cortical deformation (Soustiel et al., 2008).

*In vivo* studies employing [^11^C]PK11195 PET in rats following CCI 1 day after injury showed no differences in whole brain uptake compared to baseline values or sham-treated controls, whereas scans at 10 days showed significantly increased uptake (Folkersma et al., 2011b). In another CCI rat model, uptake of [^18^F]DPA-714 (a second-generation TSPO ligand) was observed on day 2 after injury, peaking on day 6, and remaining elevated to day 16 (Wang et al., 2014). Similar findings were seen in a weight-drop mouse model, no differences were observed in [^18^F]DPA-714 binding at day 1, but increased uptake in focal brain lesions on repeated scans at days 7 and 16 (Israel et al., 2016).

Several experimental studies have also shown that TSPO expression increases not only in the vicinity of lesioned areas but also at locations remote from sites of focal damage. Following CCI in rats, the ipsilateral thalamus and hippocampus show increased [^3^H]PK11195 binding between 3 and 14 days after injury (Raghavendra Rao et al., 2000). In the FPI model, increased uptake of [^18^F]-fluoroethyl-DAA1106 (a second-generation TSPO ligand) appears in the ipsilateral striatum up to 4 weeks' post-injury (Yu et al., 2010). In our own study, using a CCI model in rats, we found increased binding of [^123^I]CLINDE (a second-generation TSPO ligand) in contralateral motor cortex early after injury (24 h), whereas increased binding in the ipsilateral thalamus was observed first at 28 days post-injury (Donat et al., 2016).

However, TSPO up-regulation does not appear to relay any information on potential polarization pattern (Kim and Yu, 2015). In a mouse model of intracranial hemorrhage, microglia exclusively expressed TSPO and in these cells CD16/32 (M1-like) or CD206 (M2-like) were evenly distributed (Bonsack et al., 2016).

While TSPO seems a more general biomarker of glia activation, likely showing the mitochondrial changes due to the increased metabolic demand resulting from glia activation and cell proliferation (Liu et al., 2014), other targets expressed by microglia (e.g., cannabinoid type 2 receptor, P2X7 and P2Y12 receptors) could be more indicative of microglial polarization, although less tracers are available for these targets.

### Imaging microglia in humans

In humans, [^11^C]PK11195 PET has demonstrated increased TSPO expression in a variety of neuroinflammatory conditions (Owen and Matthews, 2011), including herpes encephalitis (Cagnin et al., 2001b), Alzheimer's disease (Cagnin et al., 2001a), multiple sclerosis (Banati et al., 2000), and stroke (Gerhard et al., 2005). In the normal human brain, [^11^C]PK11195 uptake is generally low, but with relatively high binding in subcortical structures, including midbrain, thalamus and putamen (Kumar et al., 2012; Kobayashi et al., 2017). The relatively increased TSPO ligand binding observed in midbrain, thalamus and basal ganglia (Kumar et al., 2012) is in keeping with the high density of microglia found in these regions in the mouse (Lawson et al., 1990) and human brain (Mittelbronn et al., 2001) in comparison to the cerebral cortex. However, increased TSPO ligand binding may reflect an increase in TSPO density rather than the number of cells expressing TSPO. These regions are highly connected through white matter to widespread brain regions. Owing to their dense connectivity, it is possible that even subtle neuronal injury, as may arise in normal aging, may result in the accumulation of secondary microglial activation in these regions, and therefore increased TSPO binding.

**Figure 2:**
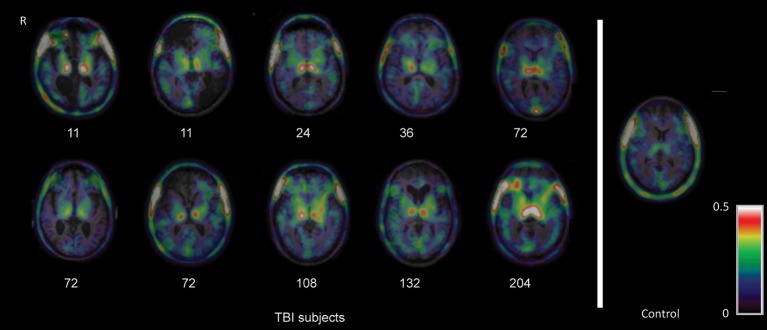
Imaging of chronic microglial activation after TBI. Images of [11C]PK11195 PET images are shown superimposed on the T1 MRI scan at the level of the thalamus for 10 TBI patients, 11 months to 17 years after injury, and a representative control participant. Numbers indicate time since injury (months). *R* right. The figure has been reproduced with permission of the copyright holder (Ramlackhansingh et al., 2011).

PET imaging with TSPO ligands provides a method to visualize microglial activity following TBI *in vivo*, although the specificity of TSPO for microglia still remains controversial. In an early study, we used [^11^C]PK11195 PET to investigate a group of 10 patients in the chronic phase 11 months to 17 years after single moderate-severe TBI (Figure 2; Ramlackhansingh et al., 2011). [^11^C]PK11195 binding potential was significantly increased in subcortical structures, particularly the thalami, putamen, and parts of the white matter. In keeping with animal work, this chronic microglial activation was seen in regions remote from focal damage and was also reduced around areas of focal injury. High [^11^C]PK11195 binding in the thalamus was associated with more severe cognitive impairment, suggesting either that microglial activation might be directly contributing to cognitive impairment or that this was a response to underlying brain injury that caused the cognitive impairment.

These initial findings have been replicated and extended in further studies. A second study by another group also used [^11^C]PK11195 PET following TBI (Folkersma et al., 2011a). They again showed increased binding in subcortical regions remote from the focal traumatic pathology, which included the thalamus, putamen, pons, and hippocampus. Increased microglial activation has also been observed in sportsmen exposed to repetitive TBI (Coughlin et al., 2015, 2017). A group of 14 active or recently retired National Football League players with a history of concussions were studied using the second generation TSPO ligand [^11^C]DPA-713. Increased binding was seen predominantly in medial temporal lobe regions in sportsmen who also showed subtle evidence of white matter damage on diffusion MRI. In our follow-up study, we used the second generation TSPO ligand [^11^C]PBR28 (Scott et al., 2015). A similar distribution of subcortical microglial activation was again seen years after single moderate/severe TBI. Prominent white matter microglial activation in areas of white matter damage was measured using magnetic resonance imaging (MRI). In addition, areas with high microglial activation showed high levels of brain atrophy over the next 6 months. This suggests that chronically activated microglia are seen in areas of persistent white matter damage, which are progressively degenerating even many years after injury.

**Figure 3:**
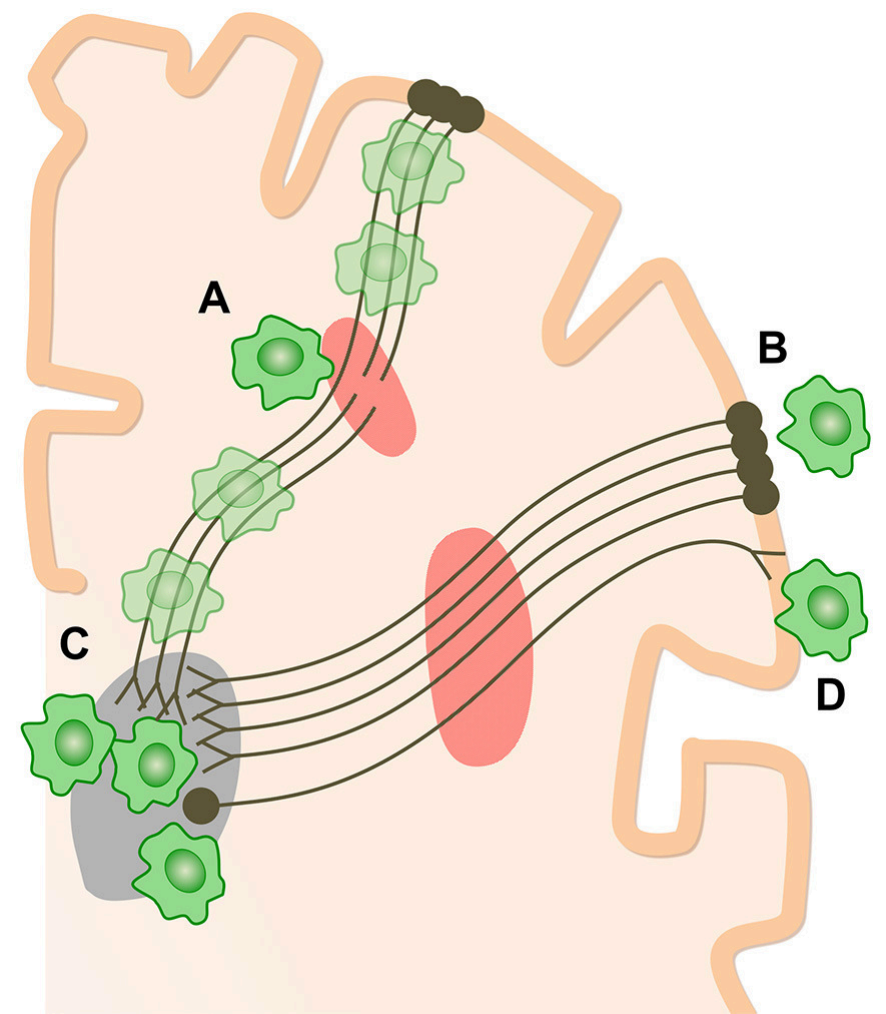
How chronic microglial activation and axonal injury may be linked after TBI. Microglial activation (green cells) and traumatic axonal injury in thalamo-cortical white matter tracts (red areas) have been demonstrated after TBI. Sites of chronic microglial activation can co-localize with axonal abnormality **(A)** as well as along the entire axonal tract affected by injury. Remote from sites of primary axonal injury, microglia may be observed both in retrograde projection areas, toward the cell bodies of damaged neurons **(B)**, and in anterograde areas **(C,D)**. The thalamus is a highly-connected structure. Thalamic microglial activation may be observed after TBI because of the high density of connections to damaged axons. The number of cortico-thalamic projections far exceeds thalamo-cortical projections. If microglial activation preferentially favors anterograde involvement, then relatively increased activation would be expected in the thalamus **(C)** compared to corresponding cortical areas **(B)**.

These studies consistently show microglial activation in regions far removed from sites of focal injuries. Whilst at first glance surprising, the observation is in keeping with the evolution of microglial activation after experimental TBI (Donat et al., 2016) and is likely to reflect slowly progressive changes within damaged white matter, in particular Wallerian degeneration (Figure 3). In our initial study, the level of thalamic microglial activation after TBI showed a strong correlation with the level of traumatic axonal injury in thalamo-cortical projections. This suggests that traumatic axonal injury might be playing a causative role in increasing chronic microglial activation (Scott et al., 2015). The effect is not specific to TBI and a similar progression of microglial activity is seen following lacunar infarcts. This suggests that as the after effects of axonal injury develop and Wallerian degeneration progresses, microglial activation might be observed in cortical and subcortical regions remote from the initial injury. A number of factors might explain the thalamic rather than cortical preponderance of microglial activation after TBI. Firstly, there is a high density of projection neurons converging in the thalamus, which might lead to a regional amplification of widespread, but sub-threshold cortical pathology (Cagnin et al., 2003). Secondly, as cortico-thalamic projections are 10 times more numerous that thalamo-cortical projections, a more intense microglial reaction to anterograde neurodegeneration might contribute to the persistent microglial reaction in the thalamus (Figure 3).

## Anti-inflammatory treatments targeting microglia activation in TBI

The relevance of microglia in the general pathophysiological response to TBI is recognized as a potential therapeutic avenue (reviewed in Chio et al., 2015) and has prompted several studies to investigate the effects of certain drugs on microglial polarization in brain injury models. Although studies in animal models indicate that microglia/macrophages respond to TBI with a transient M2 phenotype, followed by a shift to M1, and that the number of M1 cells is strongly correlated with the severity of white matter injury (Koh and DiPietro, 2011), this may differ in non-human and human primates. Therefore, therapies that prime microglia/macrophages toward the beneficial M2 phenotype after TBI may offer new anti-inflammatory strategies (Table 2). Several of these drugs, including minocycline, minozac, etanercept and the PPAR agonists fenofibrate and pioglitazone are FDA approved (Garrido-Mesa et al., 2013; Kim et al., 2014) confirming their safety and tolerability.

### Progranulin

In response to TBI, microglia/macrophages and astrocytes release inflammatory mediators with dual effects on secondary brain damage progression. The neurotrophic and anti-inflammatory glycoprotein progranulin (PGRN) attenuates neuronal damage and microglia/macrophage activation in brain injury but mechanisms are still elusive. Intracerebroventricular administration of recombinant PGRN in mice immediately before trauma reduced brain damage and neurological deficits, and restored normal levels of cytokine transcription, axonal injury and astrogliosis 5 days after TBI in granulin knockout mice (Menzel et al., 2017).

### Minocycline

Minocycline is a tetracycline derivative with anti-inflammatory and neuroprotective properties. Some of the proposed mechanisms for the anti-inflammatory properties for minocycline include the inhibitory effects on the activities of key enzymes, like iNOS, MMPs and PLA2 (Garrido-Mesa et al., 2013). In particular, it has been shown that it is able to inhibit M1 polarization state of microglia (Kobayashi et al., 2013), through the inhibition of NFκB and the interference with MAPK pathways. Minocycline significantly inhibited retinal neuroinflammation in an ischemia reperfusion (IR) model, characterized by inflammatory gene expression, leukocyte adhesion and invasion, and vascular permeability, however it failed to block neurodegeneration (Abcouwer et al., 2013). The effects of minocycline treatment in an animal model of TBI (closed head CCI in the neonate rat) revealed that the protective effects could be detected short term after injury (3 days after minocycline treatment) but not in chronic treatment (for 9 days after injury), in which microglial reactivity and neurodegeneration in all regions examined were exacerbated in minocycline-treated brain-injured animals (Hanlon et al., 2016, 2017). However, in other TBI models, such as blast injury, minocycline appears to prevent the development of neurobehavioral abnormalities (Kovesdi et al., 2012).

### Minozac

Minozac (Mzc) is an anti-inflammatory molecule that selectively reduces excessive pro-inflammatory cytokine production by activated glia toward basal levels. There is evidence that administration of Mzc in a mouse closed-head, CCI at 3 and 9 h following TBI attenuates the acute increase in proinflammatory cytokine and chemokine levels and reduces the longer-term astrocyte activation, neurologic injury and neurobehavioral deficits observed over a 28-day recovery period (Lloyd et al., 2008)

### Pharmacological inhibition of TNF-α

Pharmacological inhibition of TNF-α using etanercept has been shown to reduce the expression of microglial TNF-α in rodents subjected to FPI (Cheong et al., 2013; Chio et al., 2013) and improve the neurological outcome after stroke and TBI in humans (Tobinick et al., 2012). Etanercept also seems to be able to stimulate neurogenesis in rats (Cheong et al., 2013).

### Modulation of glutamate receptors

Although glutamate released by microglia may be related to a neurotoxic effect, it was shown that activation of metabotropic glutamate receptor 5 (mGluR5) on microglia is a novel mechanism to attenuate M1-like microglial activation and associated microglial-mediated neurotoxicity, suggesting a self-regulatory mechanism. Positive allosteric activation of mGluR5 has powerful neuroprotective effects in experimental models of CNS injury (Loane et al., 2014b).

### PPAR agonists

In traumatic brain injury, the PPARα agonist fenofibrate appears to represent a highly promising new anti-inflammatory compound. Besson et al. (2005) assessed the pharmacological role of fenofibrate in the FPI model in adult male Sprague-Dawley rats. The study revealed that the administration of fenofibrate during a clinically relevant therapeutic “time window of opportunity” at 1 h after trauma mediated a significant post-traumatic neuroprotection. This was demonstrated by improved neurological scores in the fenofibrate group at 24 h and 7 days after trauma, compared to vehicle-treated animals (Besson et al., 2005). In the case of PPARγ agonists, both pioglitazone and rosiglitazone seem to have protective effects, in particular pioglitazone reduced the histological damage and inflammation in the CCI model of TBI (Thal et al., 2011).

### HDAC inhibition

HDAC inhibitors have been found to have anti-inflammatory and neuroprotective effect in models of TBI. Scriptaid, a novel inhibitor of class I/II HDACs, was found to facilitate and enhance recovery of motor functions after CCI and protected white matter up to 35 day after TBI, as shown by reductions in abnormally dephosphorylated neurofilament protein, increases in myelin basic protein, anatomic preservation of myelinated axons, and improved nerve conduction (Wang et al., 2015). Furthermore, Scriptaid shifted microglia/macrophage polarization toward the protective M2 phenotype and mitigated inflammation. In primary co-cultures of microglia and oligodendrocytes, Scriptaid increased expression of microglial glycogen synthase kinase 3 beta (GSK3β), which phosphorylated and inactivated phosphatase and tensin homolog (PTEN), thereby enhancing phosphatidylinositide 3-kinases (PI3K)/Akt signaling and polarizing microglia toward M2. The increase in GSK3β in microglia and their phenotypic switch to M2 was associated with increased preservation of neighboring oligodendrocytes.

### Nicotinamide adenine dinucleotide phosphate (NADPH) oxidase

Nicotinamide adenine dinucleotide phosphate (NADPH) oxidase inhibitors were able to alter M1-/M2-like balance in favor of the anti-inflammatory M2-like phenotype in a CCI model of TBI (Kumar et al., 2016a,b).

## Conclusions

In recent years, there has been a growing interest in investigating the activation of microglia in TBI, because of the potential role in the progression of those patients to neurodegenerative and psychiatric diseases. The studies of microglia in animal models of TBI have allowed the manipulation of microglial numbers (by genetic or pharmacological ablation), indicating their important role in neuroprotection especially at early times post-injury, although this sequence seems to be model and species-specific. In addition, this has provided insights into the time course of the different profile of microglial activation phenotypes in the injury site and the spreading of inflammation to other areas of the brain, such as the thalamus.

*In vivo* molecular imaging of TSPO potentially provides an extremely useful biomarker of microglial activation and the effect of immunomodulatory drugs. In TBI patients, it has allowed the observation of chronically activated microglia located in areas of persistent white matter damage, even long time post injury. However, it currently only provides a one-dimensional measure, i.e., the amount of microglial activation within a particular brain region. It is clearly too simplistic to describe microglial activation *in vivo* along a single dimension (i.e., from “low activity” to “high activity”). Crucially, on the basis of current evidence, TSPO molecular imaging cannot discriminate activation phenotype, but probably reflects the (potentially uneven) summation of activity from microglia across all functional states.

In addition, there has been some debate regarding the possibility that certain TSPO ligands also recognize astrocytes or other immune cells in the brains of TBI patients. Other limitations of the application of second-generation ligands include that the binding affinities are influenced by a common polymorphism (rs6971) in the TSPO gene which causes a single amino acid substitution (A147T) in the protein (Owen et al., 2012). Because 147T TSPO binds ligands with lower affinity than 147A, this produces three classes of binding affinity across a population, which studies must therefore control for. Even after accounting for TSPO genotype, however, many second-generation TSPO ligands show high between-subject variability in uptake when using analysis methods which rely on measurement of the ligand in arterial blood (Guo et al., 2012). As for [^11^C]PK11195, high and variable plasma protein binding may be a factor (Lockhart et al., 2003). Methods of analysis such as the simplified reference tissue model (SRTM) have been developed that do not require arterial blood data, but rather use the PET imaging data from a reference region (or reference tissue) instead (Gunn et al., 1997).

To improve the interpretation of the TSPO signal, a detailed characterization is needed of how TSPO expression in humans varies with the diversity of microglial phenotypes seen *in vivo*. Future TBI work needs to provide a description of the time-course of microglial phenotype change after TBI and its relationship to TSPO expression. Novel PET ligands showing specificity for distinct functional subtypes of microglial activation would be of great utility, with cannabinoid type 2 and purinergic receptors, such as P2Y12 and P2X7, possibly providing suitable targets in the future. Another option is to combine TSPO PET imaging with different biomarkers that disambiguate the TSPO signal in a particular context, in particular neurofilament light (NFL). NFL levels in CSF have been proposed as a valid biomarker to accurately assess the level of trauma and predict the clinical outcome of the patient (Zhang et al., 2016).

Recent investigations have indicated that simple suppression of microglia activation can exert only limited beneficial effect and the inhibition of M1-like responses might be detrimental similar to a simple stimulation of M2-like phenotypes, as indicated by increased fibrosis which seems modulated by Arginase-1 in peripheral and central infection (Hesse et al., 2001; Aldrich and Kielian, 2011). In addition, pharmacological treatment with the anti-inflammatory drugs tested in several models may not have similar effects when administered in TBI patients, as seen in clinical trials for AD (Lleo et al., 2007), limiting the potential therapeutic impact. It seems therefore imperative for future studies that target microglia polarization as therapeutic strategies, to assess several markers in the target cells within a sufficient temporal window in order to show a long-term positive outcome.

## Author contributions

MS wrote most of the introduction, the manipulation of microglia in animal models and the treatments section; CD wrote most of the section on animal models of TBI and preclinical TSPO imaging in animal models, made the tables, the references and wrote part of the conclusions; GS wrote most of the part of the imaging with TSPO, made two figures and part of the conclusions; SG contributed with the image of microglia (Figure 1) and edited the manuscript.

### Conflict of interest statement

The authors declare that the research was conducted in the absence of any commercial or financial relationships that could be construed as a potential conflict of interest. All appropriate permissions have been obtained from the copyright holders of any work that has been reproduced in this manuscript.
